# Cost-effectiveness analysis of the national decentralization policy of antiretroviral treatment programme in Zambia

**DOI:** 10.1186/s12962-017-0065-8

**Published:** 2017-04-12

**Authors:** Shinsuke Miyano, Gardner Syakantu, Kenichi Komada, Hiroyoshi Endo, Tomohiko Sugishita

**Affiliations:** 1 Bureau of International Health Cooperation, National Center for Global Health and Medicine, 1-21-1 Toyama, Shinjuku-ku, Tokyo, 162-8655 Japan; 2 Department of Clinical Care and Diagnostic Services, Ministry of Health Zambia, Lusaka, Zambia; 3 Graduate School of Public Health, St. Luke’s International University, Tokyo, Japan; 4 Department of International Affairs and Tropical Medicine, Tokyo Women’s University, Tokyo, Japan

**Keywords:** Cost effectiveness, Decentralization, Antiretroviral treatment, Resource-limited settings, Zambia

## Abstract

**Background:**

In resource-limited settings with a high prevalence of human immunodeficiency virus (HIV) infection such as Zambia, decentralization of HIV/acquired immunodeficiency syndrome (HIV/AIDS) treatment and care with effective use of resources is a cornerstone of universal treatment and care.

**Objectives:**

This research aims to analyse the cost effectiveness of the National Mobile Antiretroviral Therapy (ART) Services Programme in Zambia as a means of decentralizing ART services.

**Methods:**

Cost-effectiveness analyses were performed using a decision analytic model and Markov model to compare the original ART programme, ‘Hospital-based ART’, with the intervention programme, Hospital-based plus ‘Mobile ART’, from the perspective of the district government health office in Zambia. The total cost of ART services, quality-adjusted life years (QALYs) and incremental cost-effectiveness ratios (ICERs) were examined.

**Results:**

The mean annual per-patient costs were 1259.16 USD for the original programme and 2601.02 USD for the intervention programme, while the mean number of QALYs was 6.81 for the original and 7.27 for the intervention programme. The ICER of the intervention programme relative to the original programme was 2965.17 USD/QALY, which was much below the willingness-to-pay (WTP), or three times the GDP per capita (4224 USD), but still over the GDP per capita (1408 USD). In the sensitivity analysis, the ICER of the intervention programme did not substantially change.

**Conclusion:**

The National Mobile ART Services Programme in Zambia could be a cost-effective approach to decentralizing ART services into rural areas in Zambia. This programme could be expanded to more districts where it has not yet been introduced to improve access to ART services and the health of people living with HIV (PLHIV) in rural areas.

**Electronic supplementary material:**

The online version of this article (doi:10.1186/s12962-017-0065-8) contains supplementary material, which is available to authorized users.

## Background

In 2015, a target and indicator for human immunodeficiency virus/acquired immunodeficiency syndrome (HIV/AIDS) was set in the Sustainable Development Goals (SDGs) by the United Nations (UN), government and other partners, focusing on ending the HIV epidemic by 2030 [1, 2]. To achieve this goal, UNAIDS established triple 90 (90–90–90) targets as the explicit global objective for HIV/AIDS [3]. In resource-limited settings such as sub-Saharan Africa, the decentralization of HIV/AIDS testing, treatment and care services is a cornerstone of providing universal testing, treatment and care. The geographic expansion of services from secondary and tertiary central health facilities (hospital level) to peripheral primary health facilities in rural areas (rural health centre [RHC] level) is thus an urgent need [4–6].

Lesotho was among the first countries in sub-Saharan Africa to decentralize services from hospitals to health centres via a national policy despite a severe human resource shortage in 2007 [7–9]. The development of national guidelines enabling nurses to provide ART services in health centres facilitated the successful decentralization of services [10–12]. Furthermore, the integration of HIV diagnosis, care, and treatment into primary health care services through task shifting has also been adopted as national policy in South Africa [13], Malawi [14] and Uganda [15]. Task-shifting approaches to overcome the shortage of human resources were also included in national programmes and have been shown to improve patients’ treatment outcomes and save costs in two countries [16–18]. A systematic paper also suggested that task shifting from doctors to nurses or from health care professionals to lay health workers could potentially reduce the costs of ART provision [19]. In addition, these integrative approaches were found to provide more effective outcomes including earlier enrolment in treatment [20], better adherence [15, 21, 22], better retention in treatment [15, 23–25], and higher acceptance of ART [26]. Based on these findings, a few studies have suggested that scaling up ART services to the primary care level could be a cost-effective strategy [15, 17, 27].

In Zambia, the HIV prevalence among 15- to 49-year-old adults is the seventh highest in the world (14.3%) [28]. The number of people living with HIV (PLHIV) is 980,000, 68% of whom receive ART. As Zambia is a large country, PLHIV are highly scattered, even in rural areas. The urban/rural split is approximately 60/40 [29]. Decentralization of ART services is essential to expanding service coverage, despite the severely limited resources. The Ministry of Health (MoH) in Zambia has provided HIV counselling and testing services at all health facilities nationwide since 2006 [30]. Free ART services were introduced at the hospital level in 2005 and were further expanded to some specific RHCs as a trial in 2007 through the national ‘Mobile ART Services’ programme. Under this programme, a mobile ART team comprising medical professionals such as medical doctors, nurses and pharmacists from a district hospital conducted biweekly visits to select RHCs that had been designated as ART sites; the team assisted with ART services in terms of providing human resources and building RHC staff member capacity [31]. Although the medical professionals on the team temporarily performed the drug prescription and dispensation and the laboratory services such as ordering exams and drawing blood samples at the start of the programme, these responsibilities were gradually assumed by the RHC staff under the supervision of the professionals. This programme contributed to ART service decentralization at the primary health care level and aimed to maximize the efficient use of extremely limited resources. The MoH approved this programme nationally and published the national guidelines for Mobile ART services in 2010 [32, 33]. There have been several cost-effectiveness studies on facility-based ART services in Zambia [34–39]. However, we are the first to analyse the cost effectiveness of the National Mobile ART Services Programme in Zambia to inform policy deliberations.

## Methods

### Model overview

**Fig. 1:**
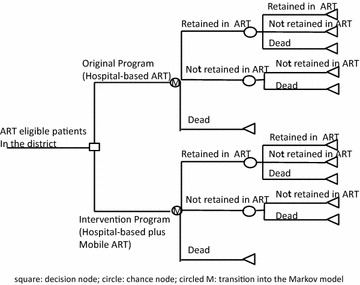
Decision tree. This decision tree was generated to compare the original programme with the intervention programme. The original programme was the ‘hospital-based ART’ services programme, and the intervention programme was the hospital-based plus ‘mobile ART’ services programme

We used a decision analytic model and conducted the analysis from the provider perspective (Fig. 1). The original programme was called the ‘Hospital-based ART’ services programme, and the intervention programme, the Hospital-based plus ‘Mobile ART’ services programme. As these services were provided in each district and managed by each district health government office (DHO), they were analysed from the DHO perspective. In the districts conducting the original programme, 1 district hospital provided ART services to an average of 6000 patients [30, 33, 40]; by contrast, in the districts implementing the intervention programme, one district hospital and an average of 5 RHCs provided ART services to an average of 7500 patients (6000 at the district hospital and an average of 1500 patients at the 5 RHCs [300 patients/centre]). We assumed that providing ART services through the Mobile ART services programme would contribute a 25% increase in ART service access (6000 → 7500 patients) based on the national ART programme reports [33, 40, 41].

We used a Markov model (Additional file 1) with half cycle corrections [42]. Our target population included PLHIV eligible for ART at the district level. The reference case was a 30-year-old patient, which reflected the median patient age in our cohort study (33.0 years old; unpublished data). As the life expectancy of a 30-year-old in Zambia was 65.0 years in 2011, a 40-year time horizon was selected for modelling [43]. The model cycle time was 1 year, since the monitoring and evaluation of the programme including patients’ treatment outcomes and retention were conducted annually by the government based on recommendations from the Joint UN Program on HIV/AIDS [44]. These parameters are shown in Table 1 [33, 40, 41, 45–48].

**Table 1:** Parameters of the decision and Markov model

Parameters	Base case in the model	Reference
Basic information		
Start age (years; age of the reference case)	30	Cohort data (unpublished)
Time-horizon (years; cycle of the Markov model)	40	Sonnenberg [42]
Transition probabilities		
Mortality rate (retained in ART → Dead) (%)	9.4	Badri [46]
Mortality rate (not retained in ART → Dead) (%)	37.5	Morgan [45]
Retention rates in the original programme (%)		
12 months after initiating ART	88.6	Cohort data (unpublished)
24 months	81.0	Cohort data (unpublished)
36 months	72.0	Cohort data (unpublished)
10 years	65.0	Assumption
20 years	60.0	Assumption
30 years	55.0	Assumption
40 years	50.0	Assumption
Retention rates in the intervention programme (%)		
12 months after initiating ART	92.6	Cohort data (unpublished)
24 months	84.1	Cohort data (unpublished)
36 months	79.0	Cohort data (unpublished)
10 years	70.0	Assumption
20 years	65.0	Assumption
30 years	60.0	Assumption
40 years	55.0	Assumption
Annual costs per person (USD)		
Original programme (hospital only)	246.45	Costing study in 2011
Intervention programme (hospital + mobile)	250.13	Costing study in 2011 (under assumption of 25% increase patients by mobile)
Utilities		
Retained in ART	0.82	Babigumira [15], WHO [47], Tengs [48]
Non-retained in ART	0.53	Babigumira [15], WHO [47], Tengs [48]

The transition probabilities between states are shown between 0 and 1, and some probabilities are time dependent (not always fixed). The reference case was a 30-year-old patient and reflected the median patient age in our cohort study (33.0 years old; unpublished data). A 40-year time horizon was selected for modelling. The model cycle time was 1 year, since the monitoring and evaluation of the programme including patients’ treatment outcomes and retentions were conducted annually by the government

### Data source

The model was developed using observational cohort data from the MoH and 15 DHOs in Zambia where the National Mobile ART services programme was implemented. We included 32,428 ART-naïve adult and paediatric patients who initiated first-line ART regimen through the programme between January 2010 and December 2011. Data were collected prospectively by clinic staff using paper-based databases and were input into computer-based databases by administrative officers. When patients died or were lost to follow-up (LTFU), i.e., they were not seen for more than 3 months after their last clinic visit, their follow-up was truncated.

### Costs

All costs are shown in Table 2. Only the operational costs of the services were calculated by considering weight of patient number, and thus programmatic costs such as monitoring and evaluation and trainings were not included in this study [47]. The costs were classified into 3 categories: (i) capital costs, (ii) recurrent costs, and (iii) drug and laboratory examination costs. All capital costs were annualized based on a 3% discount rate and estimates of useful life. All cost data were collected in the local currency (Zambian Kwacha [ZMK]) in 2011, and the total costs were adjusted to USD according to the 2011 exchange rate (1 USD = 5000 ZMK).

**Table 2:** Total annual cost of ART services

	Building	Furniture	Staff salary	Vehicle	CD4 and full blood count testing	Chemistry testing	ARV	Total in ZMK	Total in USD	Total in USD per patient
District hospital (hospital-based ART)	62,027,654.38	4,843,934.37	708,756,919.89	–	468,000,000.00	150,000,000.00	6,000,000,000.00	7,393,628,508.64	1,478,725.70	246.45
Rural health centre (mobile ART)	3,101,382.72	150,545.30	57,772,379.80	5,332,881.49	23,400,000.00	7,500,000.00	300,000,000.00	397,257,189.30	79,451.44	264.84

All cost data were collected in local currency units (Zambian Kwacha [ZMK]) in 2011, and the total costs were adjusted to USD according to the 2011 exchange rate (1 USD = 5000 ZMK)

In the original programme, 1 district hospital provided ART services to 6000 patients for 5 days/week (excepting weekends). The annual cost per patient at the hospital was calculated to be 246.45 USD. In the intervention programme, 1 district hospital and 5 RHCs provided ART services to 6000 patients at the district hospital and 1500 patients at the 5 RHCs (300 patients per centre) on 2 days/month/centre (every 2 weeks/centre). The costs of vehicles, maintenance, and fuel and the salaries of the mobile team staff were additional costs relative to the costs of ART services in the hospital. The per-patient annual cost was calculated to be 250.13 USD.

### Data analysis

The decision and Markov models were constructed using TreeAge 2014 Software (TreeAge Software Inc., Williamstown, MA, USA). Based on this model, we conducted a cost-effectiveness analysis and examined the intervention programme relative to the original programme. Both costs and effectiveness were discounted at 3% annually [49]. The model parameters including the probabilities of transition between states, costs, and effectiveness are summarized in Table 1. In the Markov model, an initial half-cycle correction was used to avoid overestimating the lifetime of the final cycle. Sensitivity analyses were also performed to examine the robustness of our results. Uncertain variables, including the mortality rate, utility scores, and costs based on different levels of Mobile ART service expansion, were entered into a one-way sensitivity analysis using programmatically plausible assumption ranges for each variable or 95% confidence intervals (Table 4). The costs were halved and doubled, and the discount rate ranged between 0 and 10%. According to the WHO guidelines, we determined that the program would be considered cost effective if the incremental cost-effectiveness ratio (ICER) was <3-fold the gross domestic product (GDP) per capita and very cost effective if the ICER was <1-fold the GDP per capita [50]. The Zambian GDP per capita in 2011 was 1408 USD, which yielded an ICER threshold of 4224 USD for our analysis [43].

### Research ethics

The authors received ethical approval from the University of Zambia Research Ethics Committee, Lusaka, Zambia (reference number 022-11-09).

## Results

### Base-case analysis

The mean annual costs per patient were 1259.16 USD for the original programme and 2601.02 USD for the intervention programme (Table 3). The mean number of quality-adjusted life years (QALYs) was 6.81 for the original and 7.27 for the intervention programme. The ICER of the intervention programme relative to the original programme was 2965.17 USD/QALY.

**Table 3:** Cost effectiveness of ART programme provision in Zambia

Programme	Cost (USD)	Incremental cost	Effectiveness (QALYs)	Incremental effectiveness	ICER (USD/QALYs)	Cost/effectiveness	Decision
Original (hospital only)	1259.16		6.81	0	0	184.78	Undominated
Intervention (hospital + mobile)	2601.02	1341.86	7.27	0.45	2965.17	357.93	Undominated

The mean annual per-patient costs were 1259.16 USD for the original programme and 2601.02 USD for the intervention programme. The mean number of quality-adjusted life years (QALYs) was 6.81 for the original and 7.27 for the intervention programmes. The cost-effectiveness ratio was higher for the intervention programme (357.93 USD/QALY) than for the original programme (184.78 USD/QALY). The ICER of the intervention programme relative to the original programme was 2965.17 USD/QALY

**Fig. 2:**
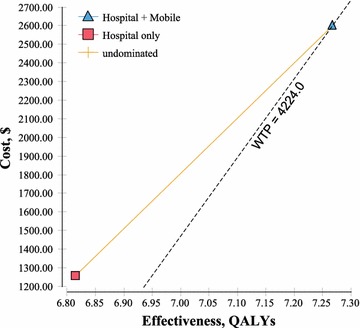
Cost-effective analysis. The cost-effectiveness graph with a willingness-to-pay (WTP) line (*dotted*). The WTP was set at 3 times the GDP per capita (4224 USD). The cost effectiveness of the original programme is plotted as a *red square* and that of the intervention programme as a *blue triangle*

Figure 2 shows the cost-effectiveness graph with the willingness-to-pay (WTP) line (dotted). The WTP was set to 3 times the GDP per capita (4224 USD). The cost effectiveness of the original programme is depicted as a red square and that of the intervention programme as a blue triangle. The WTP line intersected with the intervention programme, indicating that the intervention programme was undominated and more favourable relative to the original programme.

### Sensitivity analysis

**Fig. 3:**
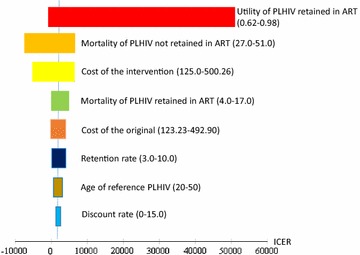
Tornado analysis. Tornado diagram showing the effects of changes in variables on the ICER

**Fig. 4:**
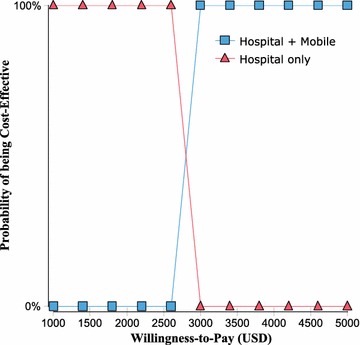
Cost-effectiveness acceptability curves. The cost-effectiveness acceptability curve shows that the original programme was always cost effective at a WTP below 2500 USD, while the intervention programme was cost effective at a WTP above 3000 USD. There was a point of indifference between the programmes at a WTP of approximately 2800 USD

The sensitivity analysis (Fig. 3; Table 4) showed that the ICER for the intervention programme relative to the original programme was most sensitive to three variables: the utility of PLHIV retained in ART, the mortality of PLHIV not retained in ART and the cost of the intervention programme. The cost-effectiveness acceptability curve is shown in Fig. 4. The original programme was always cost effective at a WTP below 2500 USD, while the intervention programme, at a WTP above 3000 USD. There was a point of indifference between the programmes at a WTP of approximately 2800 USD, which was between onefold (1408 USD) and threefold (4224 USD) the GDP per capita.

**Table 4:** One-way sensitivity analysis

	Base	Ranges (low–high)	ICER	Decision^a^	Decision threshold
Mortality of PLHIV retained in ART (%)	9.4	4.0–17.0	1768.08		11.7
			−55960.74	Dominated	
Utility of PLHIV retained in ART	0.82	0.62–0.98	−4444.94	Dominated	0.78
			1270.60		
Age of reference PLHIV (years old)	30	20–50	902.31		32.7
			−2371.56	Dominated	
Mortality of PLHIV not retained in ART (%)	37.5	27.0–51.0	1131.96		39.3
			−2038.67	Dominated	
Discount rate (%)	3.0	0.0–15.0	4878.31	Dominated	1.0
			1972.75		
Cost of the original programme (USD)	246.45	123.23–492.90	4356.33	Dominated	134.95
			182.74		
Cost of the intervention programme (USD)	250.13	125.10–500.26	92.18		304.9
			8712.78	Dominated	
Retention rate (%)	5% reduction per year	Best scenario	1644.71		–
		Original: 10% reduction/year			
		Intervention: 3% reduction/year			
		Worst scenario	4365.34	Dominated	
		Original: 3% reduction/year			
		Intervention: 10% reduction/year			

This table lists detailed data of the ICER changes and the lowest and highest values of each variable. For negative ICER values (less than zero) or those above our cost-effectiveness threshold, the final decision column indicates ‘dominated’, meaning more costly and less effective or less costly and less cost effective

*ICER* incremental cost effectiveness ratio, *PLHIV* people living with HIV, *ART* antiretroviral treatment

^a^ Dominated interventions are either more costly and less effective or less costly and less cost-effective

## Discussion

The ICER suggested that the intervention programme could be a cost-effective option for decentralization of HIV services in Zambia in addition to the original programme.

To our knowledge, this is the first study to examine the cost effectiveness of the national ART decentralization programme in Zambia. Although a similar study conducted in Uganda also examined the cost effectiveness of different modes of ART provision, it analysed a pilot programme rather than a national programme [15]. In addition, as the HIV prevalence in Uganda was half of that in Zambia (7.2 vs. 14.3%, respectively) [9], our study might have a stronger impact on the development of policies for expanding ART services in countries with a high HIV/AIDS burden, particularly those in sub-Saharan Africa. Additionally, we believe that other countries could modify and use our simple model to analyse their policies and programmes to identify an optimal approach to decentralizing ART services from an economic perspective.

We found that the intervention programme could be cost effective based on the ICER results and probabilistic sensitivity analysis. However, it was not very cost effective, since the ICER was over the GDP per capita. In developing countries including Zambia, using a stricter threshold (1X GDP per capita) could provide a more realistic scenario. The Tornado analysis showed that the ICER was largely influenced by the cost of the intervention programme; the cost factor could be addressed to make the intervention programme more cost effective than the original. According to the cost per effectiveness, our study showed that the hospital-based service cost less than the other option, and this finding was consistent with another study [15]. The intervention programme had a twofold higher annual per-person cost than the original (1259.16 vs. 2601.02 USD), although the programmes were similarly effective (6.81 vs. 7.27 QALYs). The additional costs of the intervention programme relative to the original programme included the costs of the Mobile ART services. Although the costs of buildings, furniture, facility staff, drugs, and laboratory examinations accounted for a small proportion of the total Mobile ART costs, the costs of the vehicles, maintenance and salaries of the mobile team staff might be important for controlling the total operational costs. Since these costs depend on the market price and government regulations, it could be difficult for local government officers to control the costs associated with a better programme. As Mobile ART services face greater influence from uncontrollable external factors, the long-term sustainability of these services should be further examined. However, our intervention programme had the advantages of increased access and retention despite having higher costs than the original programme, and these benefits might make the intervention programme a cost-effective option.

Although the ICER was not small enough to suggest that the intervention programme was very cost effective, to attain the UNAIDS 90-90-90 [3] objectives and universal health coverage (UHC) [51], the country still needs to decentralize HIV services to improve the service coverage and promote equal access to services.

Our findings regarding the cost effectiveness of the intervention programme were also largely influenced by the utility of the PLHIV retained in ART and the mortality of the PLHIV not retained in ART. A one-way sensitivity analysis revealed that the intervention programme was more cost effective than the original programme at a utility value over 0.78 and an annual mortality rate below 39.3%.

Healthcare workers should support patient adherence to ART and identify signs of adverse ART-related events and opportunistic infections as early as possible to avoid negative outcomes, as both these factors are normally associated with the quality of ART services; a reduction in negative outcomes could also improve the cost effectiveness of the intervention programme.

In our model, the reference case age was set to 30 years, based on the average age of our rural Zambian cohort. A one-way sensitivity analysis of age revealed that the intervention programme was expected to produce more value than the original programme for PLHIV aged less than 32 years at the time of ART initiation. This result suggests that the earlier patients are enrolled into ART, the more cost effective the intervention programme will be. This finding is consistent with the national policy in Zambia that promotes earlier HIV diagnosis, which could have a synergistic effect on the outcomes of the intervention programme.

Two patterns in the rate of retention in the Mobile ART service were examined in the sensitivity analysis. The first pattern was the best scenario, with a 10% reduction/year in the original programme and a 3% reduction/year in the intervention programme. The second pattern was the worst scenario, with a 3% reduction/year in the original programme and a 10% reduction/year in the intervention programme. Regardless of the changes in retention rate based on the best and worst scenarios, the expected value produced by the intervention did not change. This finding implied that the DHO could choose any Mobile ART service pattern to expand ART services in their district.

Our study had some limitations. The model included some assumptions; specifically, we assumed parameters such as transition probabilities, retention rates, and utilities. These assumed data might differ from the actual situation and could thus affect the results of the analysis. However, in order to develop an accurate model, we strived to obtain these data from appropriate sources such as other studies conducted in similar settings, national reports, and findings from our cohort analysis. In addition, a sensitivity analysis involving ranges of the assumed data was performed, and it did not affect the results in terms of the cost effectiveness of the programmes.

We simplified the analysis model to the greatest extent possible. Our simple model might therefore lack accuracy and details that would reflect real situations. Indicators of the severity of PLHIV, such as WHO staging and CD4 cell counts [52], were not included in the model. Adherence to ART was also not considered among the PLHIV retained in ART. Only one ART regimen, the first-line ART recommended in the latest WHO guidelines [53], was adopted in the model. Using natural history data from patients who have never been on ART to approximate the mortality of people no longer on ART could also be a limitation, as the latter might still benefit from the treatment they once received. In addition, only government health facilities such as district hospitals and RHCs were included in the model; no private facilities were included. As our target population comprised ART-eligible patients with WHO stages of 3 or 4 and CD4 cell counts <350 cells/mm^3^, and as it was based on the WHO guidelines, we decided that these factors might not affect the model analysis. It is difficult to identify people with resistant HIV who require second-line ART regimens and to identify private health facilities in our study setting of rural Zambia; therefore, these factors were not included in our model. We also wanted to develop a simplified model rather than a complicated model to allow other countries to use and modify the model to analyse their specific situations.

Regarding costs, only operational costs were calculated. The costs of training and supervision were not included because the DHO conducted these activities without separating the hospital and RHCs. The incremental annual costs also did not include the treatment costs for adverse ART-related events, opportunistic infections, second-line ART regimens for people with resistant HIV, or hospitalizations, as these events and patients were rare in our study setting. Although the approaches to ART service costs have varied in other papers [37, 54], adding these costs were expected to further improve the cost effectiveness of the intervention programme because these high costs commonly occur in the hospital.

## Conclusion

Our research findings suggest that the intervention programme (‘the National Mobile ART Services Programme in Zambia’) could be a cost-effective option compared to the original programme, in which only 1 district hospital provided ART services for an entire district. The intervention programme appears to present an optimal approach for decentralizing ART services into rural areas in Zambia, and it should be expanded to more districts where the programme has not yet been introduced to improve access to ART services and the health of PLHIV in these areas. Ultimately, the MoH of Zambia and local governments will need to exert continued efforts to maximize the benefit of this intervention by maintaining the quality of ART services.

## Additional file

**Additional file 1.** Markov Model.
