# Supplementary material for: Cost-effectiveness analysis of the national decentralization policy of antiretroviral treatment programme in Zambia
Source: Cost Eff Resour Alloc. 2017 Apr 12;15:4. doi: 10.1186/s12962-017-0065-8 (PMC5388995; doi:10.1186/s12962-017-0065-8)
Supplement: Supplementary file 1 — Additional file 1. Markov Model. [file 12962_2017_65_MOESM1_ESM.docx]

**Supplemental Appendix**

Assessment of the national decentralization policy of antiretroviral treatment programme in Zambia

*Shinsuke Miyano, Gardner Syakantu, Kenichi Komada, Hiroyoshi Endo, and Tomohiko Sugishita*

**Transition Probabilities and ART Retention Rates**

The transition probabilities and ART retention rates used in the model (Figure 1) were obtained from other published papers and assumptions based on our cohort data. An article describing the natural history of HIV/AIDS in Uganda reported an annual morality rate for patients not retained in ART of 37.5% [1]. As it was assumed that the situation was similar in Zambia, this mortality rate was used for the probability of transition to death for patients not retained in ART. Another paper analysing a cohort of HIV/AIDS patients receiving ART in sub-Saharan Africa reported an annual morality rate for patients retained in ART of 9.4% [2]. As it was assumed that the situation was similar in Zambia, this mortality rate was used for the probability of transition to death for patients retained in ART.

The ART retention rate (rate of continued ‘retained in ART’ states) was time-dependent in each scenario according to our cohort data (unpublished) and national monitoring data [3-5]. In the original program, the ART retention rates at hospitals at 12, 24, and 60 months after ART initiation were 88.6%, 81.0%, and 72.0% respectively, according to national data. However, as ART for the general population had not been provided at the hospital until 2007, there are no retention rate data beyond 60 months. Therefore, we assumed rates of 65.0%, 60.0%, 55.0%, and 50.0% for 10, 20, 30, and 40 years, respectively. In the intervention program, the combined ART retention rates at the hospital plus RHCs at 12, 24, and 60 months after ART initiation were 92.6%, 84.1%, and 79.0%, respectively, according to national data. Retention rate data after 60 months were also unavailable. Therefore, we assumed rates of 70.0%, 65.0%, 60.0%, and 55.0% at 10, 20, 30, and 40 years, respectively.

**Costs**

We conducted a costing study in 2011 at all 15 selected districts where both original and intervention programs were provided. All facilities in these districts were investigated, and final cost in each program scenario was calculated by considering weight of patient number. Only the operational costs for the services were calculated, which meant that the programmatic costs such as monitoring and evaluation and trainings were not included into this study. Data concerning the ART service-related costs in each scenario were collected by reviewing financial records and government documents at the DHOs and conducting interviews with district accounting officers. The building, equipment, vehicles and staff salary costs were obtained from national data sources, and laboratory examination and anti-retroviral (ARV) costs were estimated based on the World Health Organisation (WHO) Global Price Reporting Mechanism [6]. All capital costs were annualized based on a 3% discount rate and estimates of useful life. The cost of building space was estimated by square meters and percent use of the building space for the services with 50 years lifetime. The costs of equipment including furniture and computers were calculated by the number and frequency of being used with 5 years working life. The cost of vehicles was calculated based on the number, replacement price and percent use by the services in addition to running and maintenance costs. Staff salary was estimated by staff time of persons involved with the services.

The time and costs from the patient perspective were not included in our study. Since all the service-related costs were covered by the government, the time and costs for the patients to visit the facilities could be factors affecting the result of our economic evaluation. It was difficult to obtain and estimate that information of all patients. However, as the patients’ access to the facilities became improved by the intervention program, it was also expected that adding such factors, including less time and costs, would further improve the cost-effectiveness of the intervention program.

Figure 1. Markov Model

Markov model structure demonstrating the different health states of ART-eligible patients: (i) retained in ART, (ii) not retained in ART, and (iii) dead. The transition probabilities between states are shown between 0 and 1, and some probabilities are time-dependent (not always fixed).

**REFERENCES**

1. Morgan D, Maude GH, Malamba SS, Okongo MJ, Wagner H-U, Mulder DW, Whitworth JA: **HIV-1 disease progression and AIDS-defining disorders in rural Uganda.** *The Lancet* 1997, **350:**245-250.

2. Badri M, Bekker L-G, Orrell C, Pitt J, Cilliers F, Wood R: **Initiating highly active antiretroviral therapy in sub-Saharan Africa: an assessment of the revised World Health Organization scaling-up guidelines.** *Aids* 2004, **18:**1159-1168.

3. **Zambia National Mobile ART Services Progress Report 2010.** Lusaka, Zambia: Ministry of Health; 2011.

4. **Zambia National Mobile ART Services Progress Report 2011.** Lusaka, Zambia: Ministry of Health; 2012.

5. **Zambia National Mobile ART Services Progress Report 2012.** Lusaka, Zambia: Ministry of Health; 2013.

6. World Health Organization: **Global price reporting mechanism.** Geneva: WHO; 2011
